# Use of radiomics containing an effective peritumoral area to predict early recurrence of solitary hepatocellular carcinoma ≤5 cm in diameter

**DOI:** 10.3389/fonc.2022.1032115

**Published:** 2022-10-28

**Authors:** Fang Wang, Ming Cheng, Binbin Du, Li-ming Li, Wen-peng Huang, Jian-bo Gao

**Affiliations:** ^1^ Department of Radiology, The First Affiliated Hospital of Zhengzhou University, Zhengzhou, China; ^2^ Information Department, The First Affiliated Hospital of Zhengzhou University, Zhengzhou, China; ^3^ Vasculocardiology Department, The First Affiliated Hospital of Zhengzhou University, Zhengzhou, China

**Keywords:** hepatocellular carcinoma, peritumoral, radiomics, predictive model, early recurrence

## Abstract

**Background:**

Hepatocellular carcinoma (HCC) is the sixth leading type of cancer worldwide. We aimed to develop a preoperative predictive model of the risk of early tumor recurrence after HCC treatment based on radiomic features of the peritumoral region and evaluate the performance of this model against postoperative pathology.

**Method:**

Our model was developed using a retrospective analysis of imaging and clinicopathological data of 175 patients with an isolated HCC ≤5 cm in diameter; 117 patients were used for model training and 58 for model validation. The peritumoral area was delineated layer-by-layer for the arterial and portal vein phase on preoperative dynamic enhanced computed tomography images. The volume area of interest was expanded by 5 and 10 mm and the radiomic features of these areas extracted. Lasso was used to select the most stable features.

**Results:**

The radiomic features of the 5-mm area were sufficient for prediction of early tumor recurrence, with an area under the curve (AUC) value of 0.706 for the validation set and 0.837 for the training set using combined images. The AUC of the model using clinicopathological information alone was 0.753 compared with 0.786 for the preoperative radiomics model (P >0.05).

**Conclusions:**

Radiomic features of a 5-mm peritumoral region may provide a non-invasive biomarker for the preoperative prediction of the risk of early tumor recurrence for patients with a solitary HCC ≤5 cm in diameter. A fusion model that combines the radiomic features of the peritumoral region and postoperative pathology could contribute to individualized treatment of HCC.

## 1. Introduction

Hepatocellular carcinoma (HCC) is the sixth leading type of cancer and the second most fatal tumor worldwide (1). Among the various therapeutic options for HCC, hepatectomy and liver transplant are the primary curative treatments. However, the prognosis remains poor, with a high tumor recurrence rate of 70% after hepatectomy and 25% after transplantation (2–4). True HCC recurrence results from tumor dissemination and from the development of *de novo* tumors in the cirrhotic liver (5). In fact, in >80% of cases, tumor recurrence originates from the remnant liver, either as intrahepatic metastasis of the initial HCC or as *de novo* multicentric occurrences (6, 7). Differentiating between these two types of tumor recurrence is important for the surveillance, prevention, and management of HCC recurrence, with a cutoff of 2 years having been adopted for gross classification of early and late recurrence (8, 9).

With advances in biological and genomic technologies, predictive and prognostic molecular signatures of HCC tumors have been developed to distinguish between intrahepatic and multicentric recurrence; however, the complexity of these techniques makes their application in clinical practice difficult (10). Recent studies have reported an association between aggressive pathological factors, such as a high tumor grade, microvascular invasion (MVI), and microsatellite lesions, and tumor recurrence within 2 years after HCC surgery (11, 12). Currently, the pathological signature of tumors likely to recur is determined using postoperative and histopathologic information. Although these features can be obtained by biopsy prior to surgery, preoperative biopsy is not recommended in routine practice owing to the risk of tumor seeding, as well as the inconsistency of biopsy findings with the final pathological findings after tumor resection (13, 14). If early recurrence of HCC could be predicted preoperatively, appropriate treatment could be provided, including avoiding unnecessary surgery (15).

Medical imaging is an integral component of the routine management of patients with HCC. As imaging closely correlates with histopathologic traits of the tumor, some imaging features may reasonably predict early HCC tumor recurrence (16–18).

Radiomics refers to the conversion of medical images into high throughput quantifiable features, with the technique having greatly advanced the development of precision medicine (19, 20). Radiomics can capture the three-dimensional information of the total tumor, acting as a virtual whole tumor biopsy (21, 22). As such, the technique can effectively characterize tumor heterogeneity. Moreover, from a pathological perspective, peritumoral parenchyma, which is rich in highly invasive cells and prone to microvascular invasion (MVI) and satellite nodules which promote MVI and metastases, is a representative feature of cancerous heterogeneity (23, 24). Recently, a few studies have employed radiomics to predict early recurrence of HCC after tumor resection (25, 26). Noteworthy, these previous studies solely extracted engineered features from within tumor annotations. As the peritumoral area harbors highly invasive tumor cells, radiomic analysis of changes in the peritumoral area might hold prognostic information. Accordingly, our aim in this study was to investigate whether peritumoral radiomic analysis, using contrast-enhanced computed tomography (CT), could improve the prediction of HCC recurrence for patients with solitary HCC tumor ≤5 cm. As well, we determined the appropriate peritumoral distance to increase diagnostic performance. To evaluate the added value of radiomics for the prediction of HCC recurrence, we compared the preoperative histological models developed with conventional models based on postoperative pathology.

## 2. Materials and methods

### 2.1. Statement of ethics and study sample

Our study was approved by our hospital’s research ethics board. The requirement for informed consent was waived owing to the retrospective design of our study.

**Figure 1:**
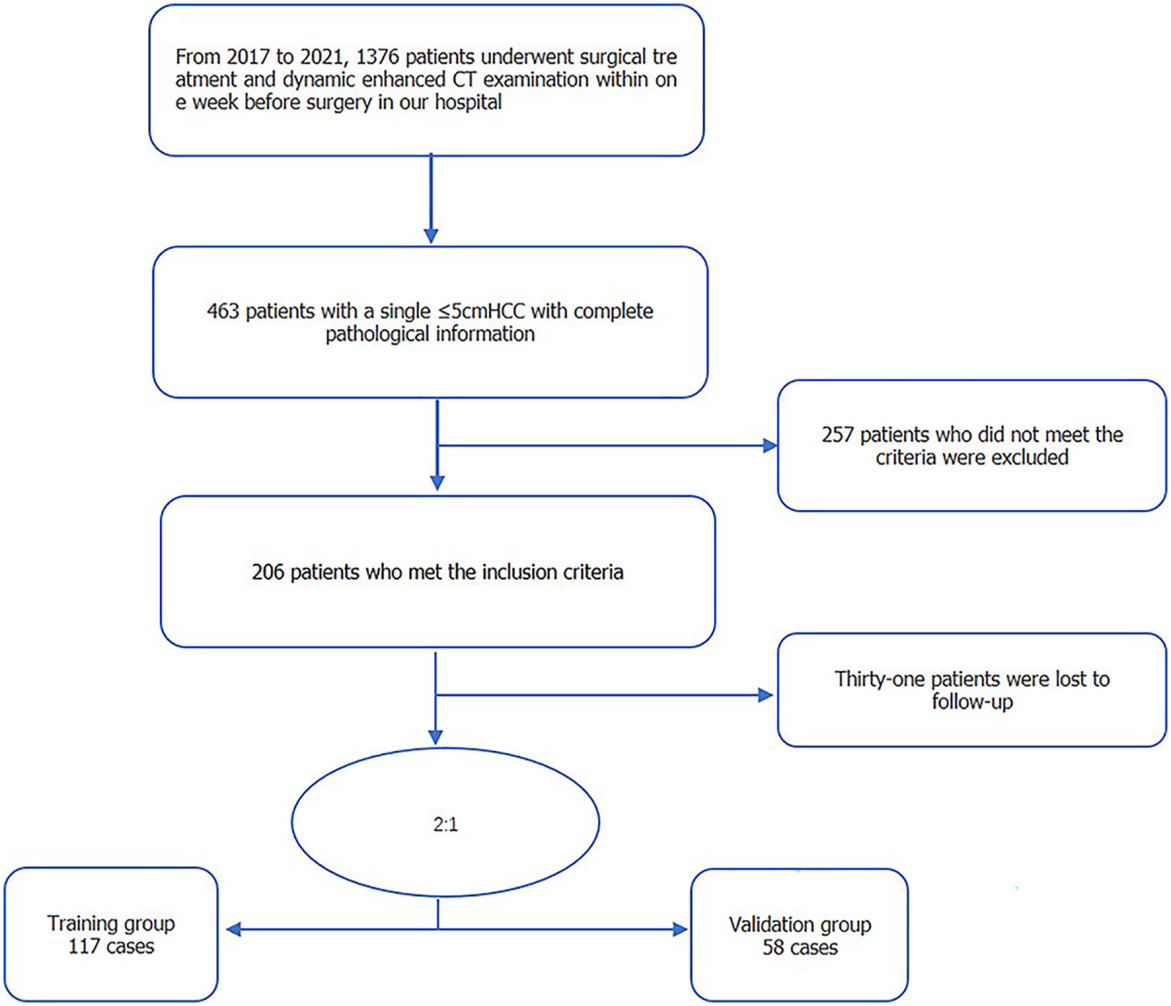
Flow chart of the screening of patients in the study group for early tumor recurrence.

Eligible for our study were the 175 patients with isolated HCC treated at our hospital between 2017 and 2021 (**Figure 1**). The inclusion criteria were as follows: solitary HCC tumor, with the longest diameter ≤5 cm; absence of extrahepatic metastasis or major vascular invasion on preoperative imaging; no prior history of HCC-related treatments; complete histopathologic description of HCC; and enhanced CT with sufficient image quality, obtained within 1 week before surgery. Excluded were patients with: a follow-up of <2 years; presence of infiltrative type HCC, which makes it difficult to judge the tumor edge; presence of other malignant tumors; and incomplete clinical baseline data. Clinical baseline information of all enrolled patients, including age, sex, and laboratory tests, was retrieved from patients’ electronic medical records.

### 2.2. CT imaging protocol and image analysis

CT imaging was performed using a Discovery 750 HD (GE Medical, USA), SOMATOM Force (Siemens Medical, Germany), Brilliance ICT (PHILIPS, Netherlands), or Aquilian One 640 (Toshiba, Japan) CT systems. Images were obtained using a tube voltage is 120 kV, a scanning layer thickness of 5 mm and between-layer spacing of 5 mm, with automatic pitch matching. Contrast agent (iopromide, 370 mg/mL, GE Medical Systems, 1.5 mL/kg) was injected through the right anterior median cubital vein using a high-pressure syringe, at a rate of 3 mL/s, followed by a 20 ml physiological saline rinse. When the contrast in the descending aorta reached 100 HU, the small-dose trigger technique was adopted, with the arterial phase images collected at a delay of 10 s and the portal vein phase images at a delay of 30 s. Patients were instructed to hold their breath during CT enhanced image capture to avoid the image inconsistency phenomenon during the arterial and portal vein phase. Coronal and sagittal plane images were reconstructed to yield a three-dimensional image.

**Figure 2:**
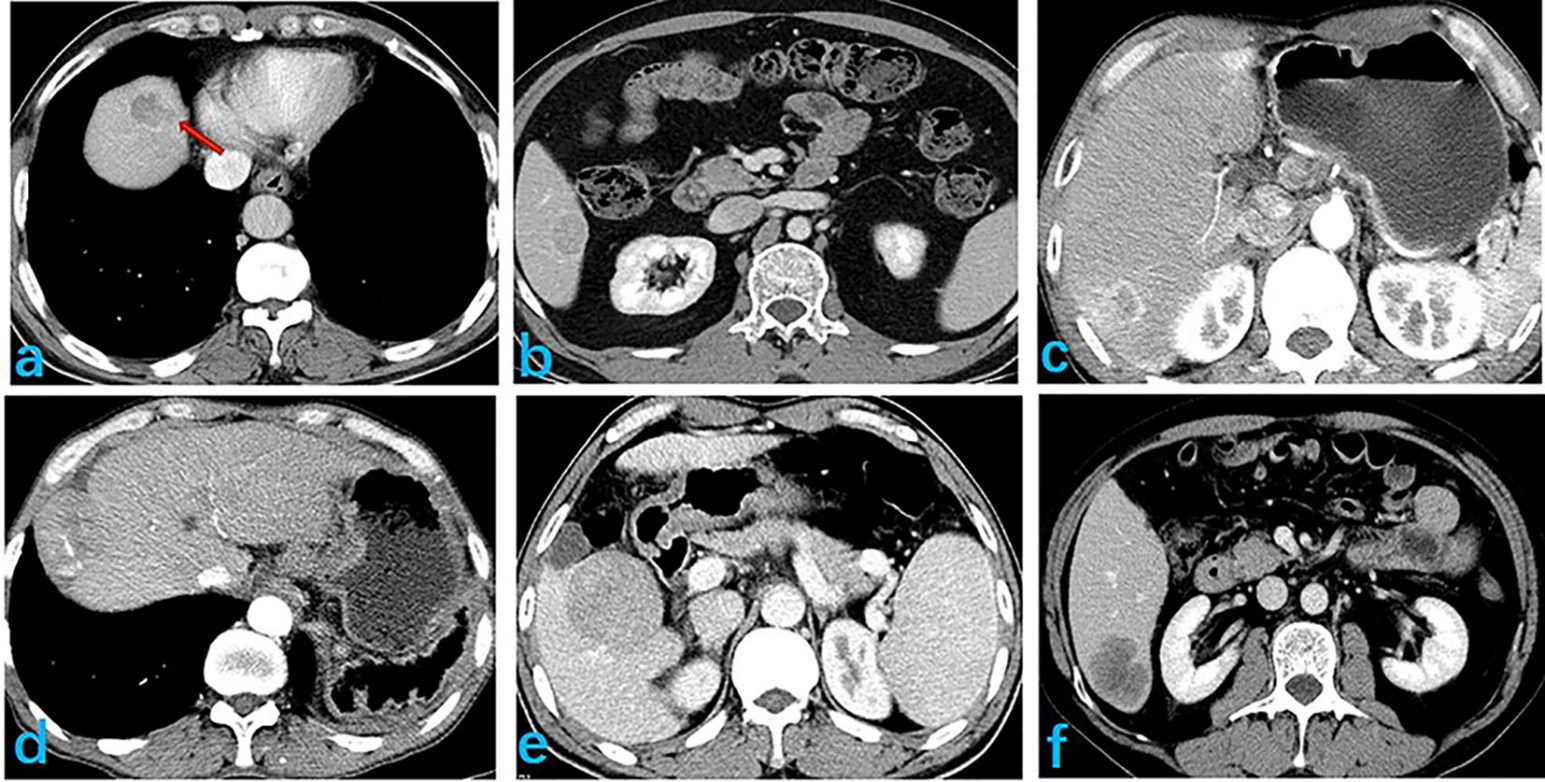
Selected image features extracted, showing: **(A)** smooth tumor edge; **(B)** complete envelope; **(C)** peritumor enhancement; **(D)** discontinuous intratumoral artery; **(E)** peritumoral low density ring; and **(F)** clear differences at the liver-tumor interface.

Tumor features on the images were assessed by two experienced physicians, with 10 and 15 years of experience in image-based diagnosis of liver disease. These assessments were performed with blinding to pathological results. Disagreement regarding features between the two physicians were resolved by discussion and consensus. The following features were evaluated on images (**Figure 2**): tumor size; characterization of the tumor margin as smooth or not smooth; presence, absence, or incomplete tumor envelop; peritumoral enhancement; and presence or absence of an intratumoral artery, low-density loops, liver-tumor interface difference, RVI, and two-trait predictor of venous invasion (TTPVI). Positive RVI was defined by the presence of intratumoral arteries and the absence of low-density loops and a liver-tumor interface difference, with a positive TTPVI defined by the presence of intratumoral arteries and the absence of low-density loops around the tumor.

### 2.3. Radiomic feature analysis

#### 2.3.1. Image preprocessing

**Figure 3:**
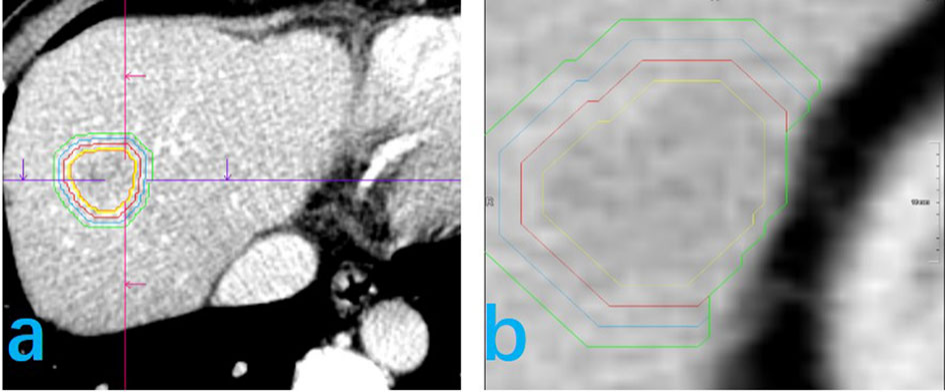
Delineation of the volume of area of interest (VOI) for feature extraction is shown for two patients **(A, B)**. The yellow line represents the tumor boundary, the red line the 5-mm physical expansion of the peritumor area, the blue line the 10-mm expansion, and the green line the 15-mm expansion. In this study, the 5-mm and 10-mm peritumor areas are discussed. **(B)** The peritumoral expansion ends at the edge of the liver.

CT image reconstruction was performed using a standard algorithm, with a 1-mm layer thickness and 1-mm spacing between layers. The window width was set to 220 HU and the window position to 40 HU (**Figure 3**).

#### 2.3.2. Delineation of the volume area of interest

Reconstructed images were stored in DICOM format, transferred into Siemens Syngo Research Frontier platform, and then entered into the Radiomics software (version 2.6) for VOI determination. The semi-automatic general segmentation method was used to outline tumors (**Figure 3**). A radiologist, with 10 years of experience in image processing, verified the area of interest for each level of the delineated range, manually correcting the area, as needed, to obtain the tumor VOI (Vtumor). The Vtumor was then confirmed by another radiologist, with 15 years of experience. Once the Vtumor was confirmed, the area was expanded in all three dimensions, using an automatic expansion algorithm. When the Vtumor+peritumoral crossed blood vessels, gas, bone, a bile duct, or exceeded the liver edge, the contour was manually modified to obtain a Vtumor+peritumoral. As such, the Vtumor+peritumoral is not a uniform volume. Here, we got Vtumor+5mm, Vtumor+10mm, Vtumor+15mm.The resultant VOI obtained in either the arterial or portal vein phase was automatically matched to the other phase to ensure correspondence of the tumor volume and boundary in both phases. The consistency of VOI delineation was evaluated by repeating the process for 50 patients, randomly selected, at a 1-month interval, with volumetric delineation of the region of interest performed by the same radiologist or another radiologist also with 10 years of experience to obtain a stable radiomics feature.

#### 2.3.3. Feature extraction and model building

After delineation of the VOI, all features of the Radiomics software were selected for computation of the features within the Vtumor+peritumoral volume during both the arterial and portal vein phases. In total, 1691 parameters were computed for each phase, including morphological, first order, texture, filter transform, and wavelet transform features. Dimensionality reduction of the data is the first step in building a radiomics model. In this study, we used a variety of methods to identify the most stable and correlated features to reduce the dimensionality of the data. First, stable features were extracted using images from 50 randomly selected cases. Inter- and intra-group correlation coefficients were calculated and features with a correlation coefficient >0.80 were selected as features having high stability. Second, Spearman’s correlation was used to remove correlated features. Third, a t-test or Mann–Whitney rank sum test was used to select the features which correlated with early tumor recurrence, based on postoperative clinical and pathological data. A LASSO algorithm was then used to create the radiomics model.

The LASSO algorithm sets the constraints to reduce the dimension of the existing data by compressing the data coefficients which have little or no influence in the model to ‘0’ through a penalty function and to set the sum of the absolute values of the regression coefficients to be less than a constant. Subsequent to the LASSO procedure, the robustness of the model can be defined by an equation with weighted regression coefficients. The total calculated value of coefficients yields the Radscore.

### 2.4. Statistical analysis

Cases were randomly allocated to either of the two following groups, a model training and a model validation group, using SPSS. The Kolmogorov–Smirnov test was used to evaluate the normality of the distribution of the data. To evaluate between-group differences, Student’s t-test was used for normally distributed data and a Mann–Whitney U test for a non-normal distribution. Chi-squared or Fisher’s tests were used to compare categorical data. The clinical baseline data were analyzed and the risk factors for early recurrence were identified using univariate and multivariate logistic regression analyses. A receiver operating curve (ROC) analysis was used to evaluate the predictive efficiency of different radiomics models, with the area under the curve (AUC) as a primary performance metric. Delong’s test was used to compare between different models to predict early postoperative recurrence of HCC, with a P-value <0.05 considered as significant. Decision curve analysis was used to compare the clinical value of the different models. A calibration curve and Hosmer-Lemeshow test were used to evaluate the calibration capability of the selected model.

All analyses were performed using R software package (version 3.6.1, http://www.R-project.org).

## 3. Results

### 3.1. Patient characteristics

The study group included 175 patients with HCC, 152 men and 23 women, with a median age of 55 (range, 31–72) years. Of these, recurrence occurred within 2 years was identified in 56 patients (early recurrence), with no recurrence within 2 years in the other 119 patients. For model development, 117 patients were randomly allocated to the model training group, including 100 men and 17 women, median age of 53 (range, 31–71) years, 22 with early recurrence and 95 without recurrence. The model validation group included 58 patients, including 52 males and 6 females, median age of 56.5 (range, 34–72) years, with early recurrence in 21 patients and no recurrence in the other 37. The baseline patient data for all groups are reported in **Table 1**. There were no differences in the distribution of clinical indicators and image features between the training and validation groups.

**Table 1:** Baseline data table of 175 hepatocellular carcinoma patients who met inclusion criteria.

Characteristics	training(n=117)	validation(n=58)	Z /χ²value	*P* value
Age(years)				
median(IQR)	53.00(48.00,60.00)	56.50(51.00,61.00)	-2.270	0.023
range	31.00-71.00	34.00-72.00		
gender				
male	100	52	0.595	0.441
female	17	6		
AFP (ng/ml)				
<20	61	28	0.231	0.631
≥20	56	30		
pathology grade				
I-II	95	42	1.760	0.185
III-IV	22	16		
capsular invasion				
yes	9	6	0.348	0.555
no	108	52		
tumor size				
≤2cm	20	12	0.336	0.562
>2cm	97	46		
hepatitis B virus				
yes	95	52	2.064	0.151
no	22	6		
hepatitis C virus				
yes	7	0	2.225	0.136
no	110	58		
liver cirrhosis				
yes	83	41	0.001	0.973
no	34	17		
Buga				
yes	5	2	0.000	1.000
no	112	56		
preoperative ALT				
≥40	33	24	3.064	0.080
<40	84	34		
preoperative AST				
≥40	32	24	3.507	0.061
<40	85	34		
liver function				
A	108	52	0.348	0.555
B-C	9	6		
hepatic encephalopathy				
yes	0	0		
no	117	58		
ascitic fluid				
yes	6	5	0.319	0.572
no	111	53		
bilirubin				
<25	105	47	2.576	0.108
≥25	12	11		
albumin				
35-55	90	47	0.386	0.535
<35or>55	27	11		
prothrombin time prolonged				
4-6	0	0		
>6	117	58		
peritumoral enhanced				
yes	21	10	0.013	0.908
no	96	48		
smooth margin				
yes	59	32	0.350	0.554
no	58	26		
complete pseudocopula				
complete	57	29	0.223	0.894
incomplete	48	22		
no	12	7		
intratumoral arteries				
negative	81	40	0.001	0.971
positive	36	18		
peritumoral low density				
negative	86	43	0.008	0.929
positive	31	15		
tumor-liver differcence				
clear	87	42	0.076	0.783
unclear	30	16		

### 3.2. Univariate and multivariate analysis of clinical indicators and imaging features

The probability of early recurrence was 32% (56/175), overall. Results of univariate and multivariate logistic regression analyses relating clinical indicators and image features with pathological outcomes are presented in **Table 2**. Pathological tumor grade and MVI were significantly correlated with early recurrence, but with no significant correlation between early recurrence and peritumoral enhancement and tumor pseudocapsule integrity. The postoperative tumor pathological grade, MVI, and peritumoral enhancement were independent risk factors for early postoperative tumor recurrence.

**Table 2:** 175 patients univariate and multivariate logistic regression analysis.

variant	univariate			multivariate	
	OR (95%CI)	*P* value		OR (95%CI)	*P* value
**gender**					
male vs female	0.833(0.290,2.397)	0.735			
**Age**(years)					
≧60 vs <60	1.036(0.993,1.082)	0.104			
**AFP(ng/ml)**					
<20 vs ≧20	1.835(0.911,3.699)	0.089			
**pathology grade**					
I-II vs III-IV	0.212(0.098,0.460)	0.000*		0.285(0.123,0.660)	0.003*
**capsular invasion**					
no vs yes	1.605(0.517,4.987)	0.413			
**tumor size**					
≦2cm vs >2cm	0.832(0.332,2.087)	0.695			
**hepatitis B**					
no vs yes	1.604(0.570,4.515)	0.371			
**hepatitis C**					
no vs yes	0.826(0.354,1.927)	0.658			
**cirrhosis **					
no vs yes	0.933(0.440,1.979)	0.856			
**buga**					
no vs yes	1.239(0.232,6.629)	0.802			
**preoperative ALT **					
≦40U/L vs >40U/L	1.000(0.991,1.009)	0.989			
**preoperative AST**					
≦40U/L vs >40U/L	1.002(0.990,1.014)	0.750			
**liver function**					
A vs B-C	2.239(0.485,10.347)	0.302			
**hepatic encephalopathy**					
no vs yes					
**ascitic fluid **					
no vs yes	1.832(0.509,6.587)	0.354			
**albumin**					
35-55μmol/L vs <35or>55μmol/L	0.977(0.922,1.037)	0.447			
**peritumoral enhanced**					
yes vs no	5.345(2.490,11.477)	0.000*		2.587(1.048,6.390)	0.039*
**Smooth edge**					
yes vs no	0.827(0.421,1.625)	0.581			
**complete pseudocapsule **					
yes vs incomplete and no	0.460(0.225,0.940)	0.033*			
**intratumoral arteries**					
negative vs positive	1.920(0.938,3.932)	0.074			
**peritumoral low density**					
negative vs positive	0.953(0.434,2.092)	0.904			
**tumor-liver differcence**					
clear vs unclear	0.573(0.272,1.207)	0.143			
**MVI**					
negative vs positive	4.525(2.186,9.365)	0.000*		2.707(1.150,6.372)	0.023*

*means p < 0.05.

### 3.3. Selection and modeling of radiomic features

**Figure 4:**
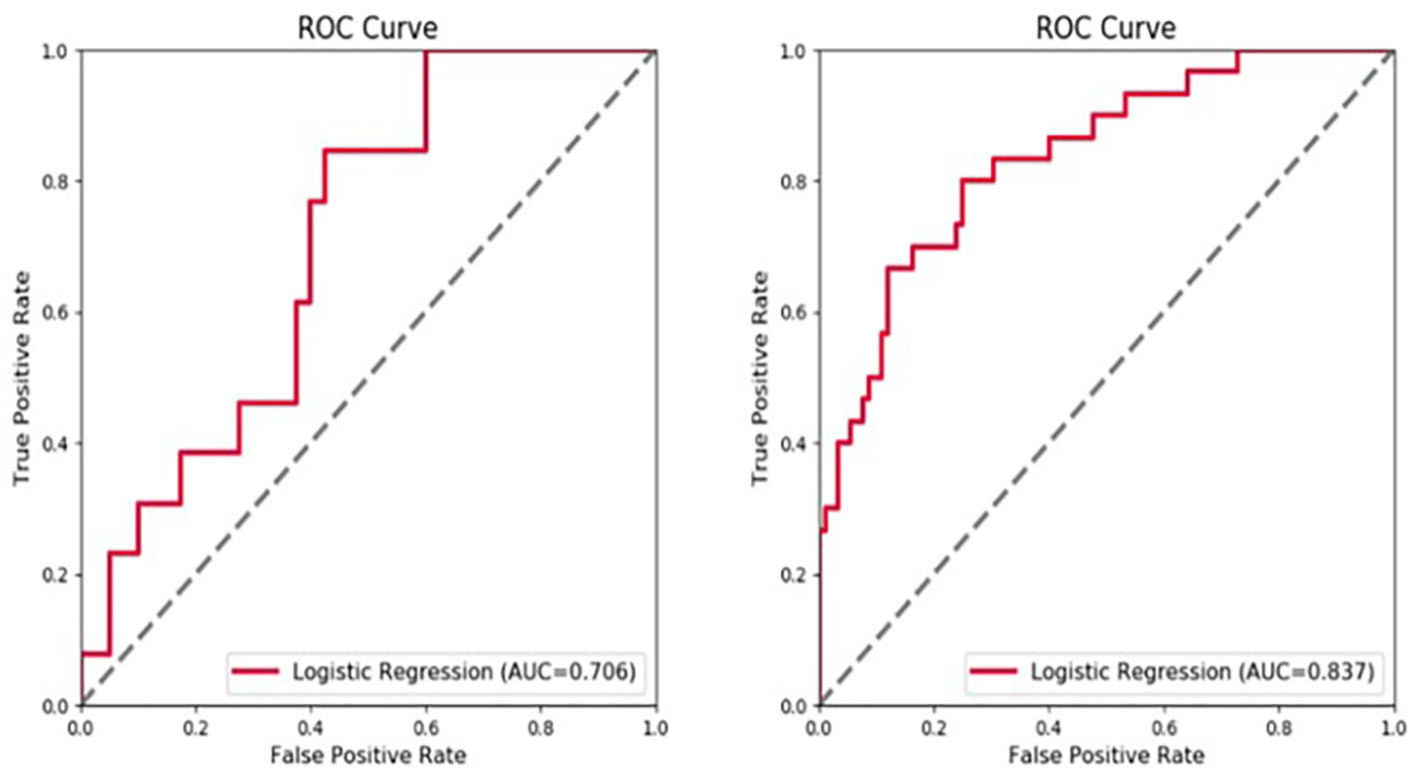
Receiver operating characteristic (ROC) curve showing the predictive value (area under the curve, AUC) for the 5-mm peritumoral area for early tumor recurrence: combined arterial and portal venous phase, AUC of 0.706 for the validation set and 0.837 for the training set.

In a first step, tumor and radiomic features of the peritumoral area at 5-mm and 10-mm were used as separate models, with both tumor and peritumoral features being predictive of early recurrence. The tumor features had the highest predictive value in both the arterial and portal venous phase. The AUC values for the radiomics models at a peritumoral distance of 5 mm and 10 mm in the validation set were not significantly different, with values of 0.620 and 0.617, respectively, for the arterial phase and 0.644 and 0.610, respectively, for the portal vein phase. Therefore, assessment of peritumoral features at a distance >5 mm from the tumor did not improve predictive value of the model. For the 5-mm VOI assessed as a whole unit, adding the peritumoral radiomic features improved the prediction efficiency of the model. Moreover, the model combining the features in the arterial and portal phases was superior to the single-phase model (**Figure 4**).

### 3.4. Comparison of preoperative peritumoral radiomics model to the postoperative clinicopathological model

The postoperative pathological grade and MVI were independent risk factors for early postoperative recurrence. Peritumoral enhancement on preoperative image was also an independent predictor of early postoperative recurrence. The AUC for the prediction model including these three indicators was 0.753 (95%CI, 0.721, 0.845), compared to 0.786 (95%CI, 0.684, 0.816) for the model including the preoperative peritumoral radiomics only. Therefore, there was no difference in predictive value between these two models (Delong test, P=0.586), indicative that the preoperative peritumoral radiomics prediction model can achieve comparable results to postoperative pathology (**Table 3**).

**Table 3:** Preoperative model and postoperative clinicopathological model.

Model	Sens	Spec	PPV	NPV	Acc	AUC
preoperative Radiomics	0.231	0.976	0.800	0.750	0.603	0.786
postoperative clinicopathological	0.326	0.939	0.636	0.811	0.633	0.753

### 3.5. Development of a preoperative predictive model including both peritumoral and postoperative clinicopathological features

**Figure 5:**
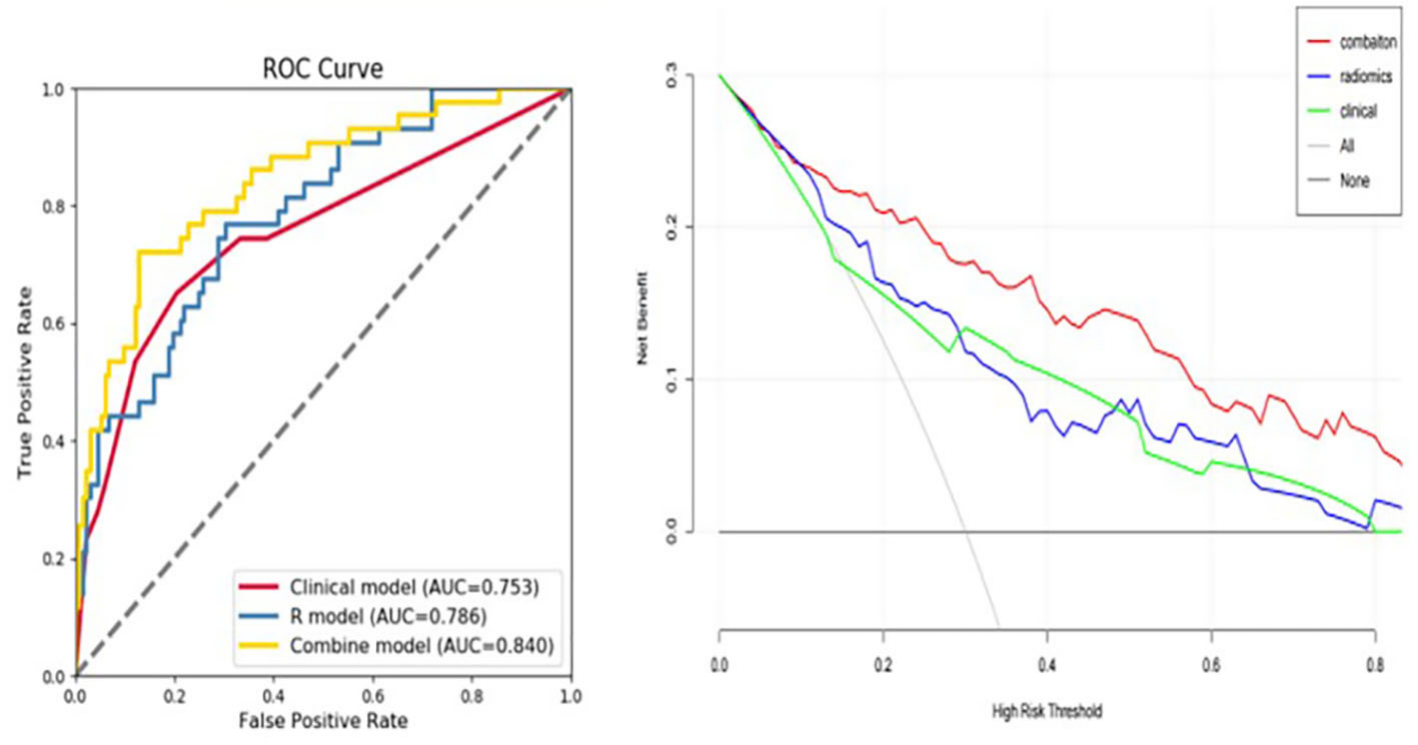
Predictive value of developed models used as an independent diagnostic index of early tumor recurrence. The area under the curve (AUC) value for the fusion model, combining the 5-mm peritumoral area and clinicopathological information was 0.840, indicating a higher practical value than either model alone, with AUC values of 0.786 and 0.753, respectively.

**Figure 6:**
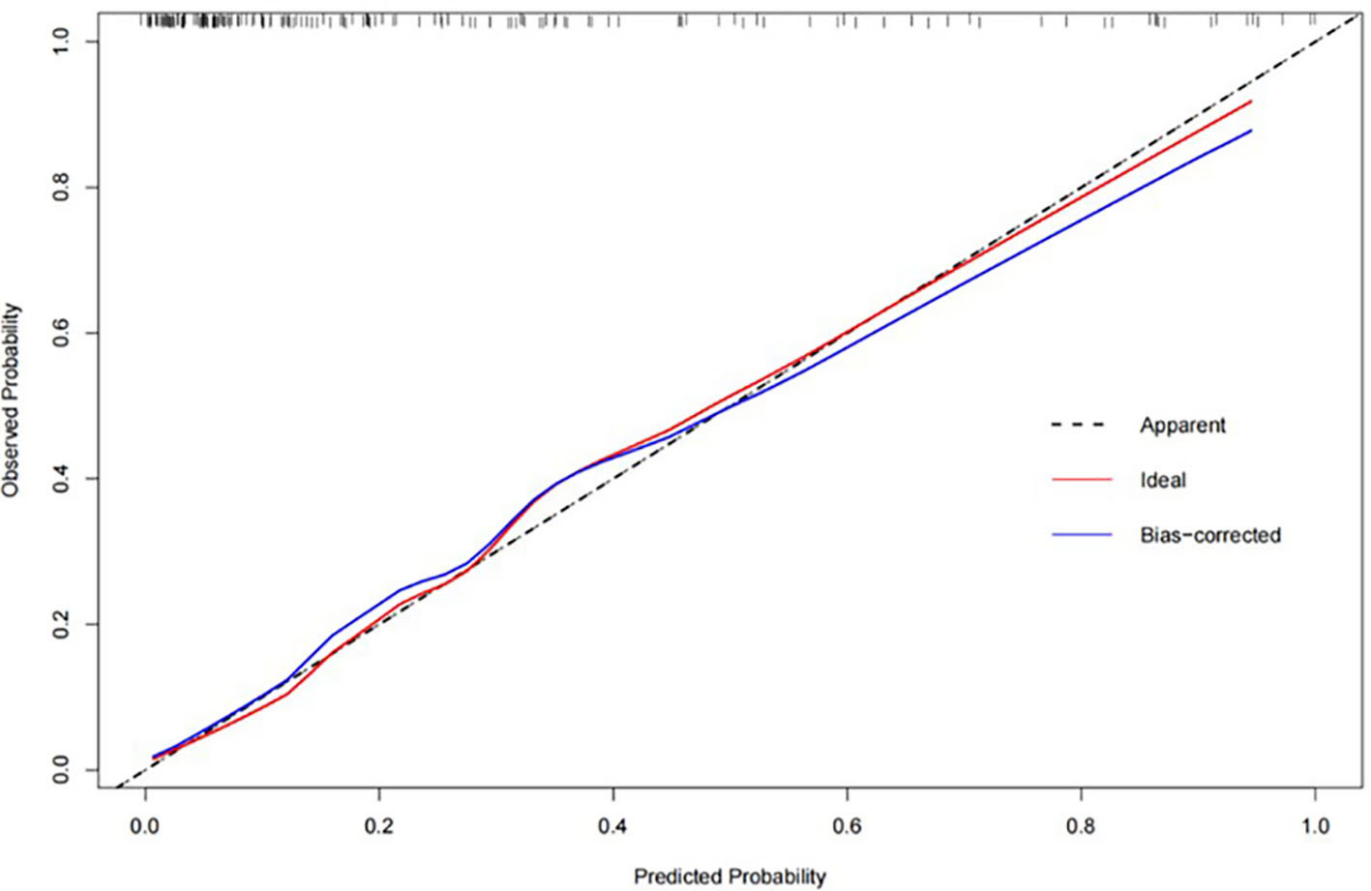
The calibration curve for the fusion model, combining the combining the 5-mm peritumoral area and clinicopathological information, showing a high consistency between predicted early postoperative tumor recurrence and actual recurrence in our study population.

The fusion model which incorporates both the preoperative peritumoral radiomics features and the postoperative pathological parameters yielded a higher diagnostic and predictive efficacy, with an AUC of 0.840, compared to a value of 0.753(95%CI,0.721-0.847) for the clinicopathology model and 0.786(95%CI,0.684-0.816) for the 5-mm peritumor model combining the dynamic arterial and portal vein phase (**Table 3**; **Figure 5**; Hosmer-Lemeshow test, P=0.536). The prediction of the fusion model fits well with real outcomes, with the calibration curve showing high consistency between the fusion model for the whole study population and actual early postoperative tumor recurrence (**Figure 6**).

## 4. Discussion

In this study, we established and validated a preoperative model based on enhanced CT radiomics that incorporated the peritumoral area for an individualized prediction of the early recurrence of HCC in patients with a tumor size ≤5 cm. Our findings indicate comparable performance between the preoperative model and postoperative pathology. Therefore, the CT image-based preoperative model can facilitate clinical decision-making, such as the selection of appropriate recipients for liver transplant as a curative treatment for HCC. We further indicate that enhancement features of a 5-mm peritumoral area was sufficient, yielding the same predictive efficiency as a 10-mm area. Lastly, we show that a fusion model, which combines radiomic features of the 5-mm peritumoral area and pathological information provided good agreement with actual early recurrence in our study population and, thus, yielded had a good clinical practice value. The good predictive outcomes of our image-based preoperative model are of clinical importance considering the high early tumor recurrence rate after resection or liver transplantation for the treatment of early stage HCC, calculated as 32% (56/175 patients) in our study population.

Early-stage HCC is heterogeneous, with diverse clinical outcomes. Recent studies have demonstrated that radiomics signatures could be used for diagnosis, prediction, and prognostic evaluation at the molecular level. The good predictive efficiency of our preoperative radiomics model of the 5-mm peritumoral area is consistent with this view and could guide clinical protocol development for HCC management. Studies on the peritumoral microenvironment are increasing (27, 28), with changes in the peritumoral microenvironment captured by radiomic features having been shown to efficient in predict benign and malignant pulmonary nodules, lymph node metastasis of lung cancer, pathological classification of breast cancer, and glioma recurrence (29–31). In our study, the addition of peritumoral radiomics to the preoperative model yielded the same predictive power as the clinicopathological model.

We identified pathological grade, MVI, and peritumoral enhancement as independent risk factors for early recurrence, which is consistent with findings from previous studies (32–34). Peritumoral enhancement is caused by abnormal peripheral perfusion, which may be caused by physical extrusion or MVI. MVI as an independent risk factor for HCC recurrence has been shown in many studies (35, 36). The pathological tumor grade has also been shown to be closely related to prognosis, with no significant difference in overall survival time between patients with grade I or II tumors, as well as between patients with grade III and IV tumors (37, 38). Accordingly, to evaluate the predictive efficiency of our models, we considered grades I and II as one group and grades III and IV as the other.

Previous studies regarding early postoperative recurrence of HCC have focused on larger tumors, with a diameter ≥5 cm identified as an independent risk factor for early recurrence (39); tumors with a diameter ≤5 cm have not been sufficiently considered in previous studies. ‘True’ recurrence is generally considered as tumor recurrence that occurs within the first 2 years after curative treatment for HCC as these recurrent tumors are generally caused by the invasive characteristics of the primary tumor. The temporal and spatial heterogeneity of HCC increases the difficulty of grasping the full biological characteristics of these tumors (40). In clinical practice, the reliance on pathological information to describe the biological characteristics of HCC is limited by tissue sampling techniques which may be insufficient for the heterogeneity of these tumors. Our findings show that radiomics, which capture three-dimensional information of the tumor, can provide a solution to the limitation of sampling for pathological outcomes (41, 42). A previous study on colorectal cancer prognosis showed that CT features extracted from three-dimensional segmentation are superior to maximum cross-sectional segmentation in predicting survival (43). In our study, inclusion of peritumoral data provided a more comprehensive view of the biological characteristics of HCC, yielding good predictive efficiency of a preoperative model. Chong et al. (44) similarly reported that multi-scale and multi-parametric radiomics of gadoxetate disodium–enhanced magnetic resonance (MR) images provided good prediction of outcomes among patients with a solitary HCC of ≤5 cm. They also compared the efficiency of including tumor+5 mm and tumor+10 mm areas, showing that a 5 mm area was sufficient, which is consistent with the findings of our study. Of note,this study (44) have reported on the superior prognostic efficiency of a Vtumor+10 mm area did not study 5-cm tumors individually.In our radiomics model, wavelet features were more heavily weighted, especially in the peritumoral area features are more likely related to effects of recurrence on the roughness of the tumor contour.In addition, the_square_ngtdm_Coarseness and original_shape_SphericalDisproportion features, which reflect tumor growth in more detail, also contributed to our model. In the final model, the wavelet-LHL_glcm_Contrast feature in atrial phase is heavier in the recurrence group, it reveals tumor heterogeneity measured in the dynamic arterial enhanced CT phase, and this is consistent with other study (45). These radiomic features, related to tumor biology and heterogeneity, complement the visual image content.

Currently, there are no proven adjunctive therapies to reduce the risk of liver cancer recurrence after resection. However, patients at a high risk of recurrence are potential candidates for clinical trials on adjuvant therapy. The risk of recurrence also influences the surgical treatment strategy selected, with primary tumor resection and liver transplant being considered as the most effective strategy to prevent recurrence, with salvage liver transplant considered very suitable approach in the event of recurrence. However, personalized preoperative prediction of recurrence risk has remained challenging and, thus, information to guide surgical strategy lacking. Our preoperative radiomics model could provide a novel solution to this clinical challenge, with good agreement with the postoperative pathology supporting its use of personalized treatment decisions. As examples, patients at low risk of tumor recurrence may not require adjuvant therapy and may require less intensive follow-up monitoring. By comparison, patients at high risk of tumor recurrence would be candidates for adjuvant therapies and intensive follow-up for up to a period of 5 years post-surgery.

The limitations of our study need to be acknowledged in the interpretation of our results for practice. Foremost is the inherent bias on outcomes introduced by the retrospective design using data from a single center. A larger cohort population and external validation are warranted to support the use of our model in practice. Second, the sample was limited, with only 175 patients included and with the majority of these patients presenting with hepatitis B-related HCC. The representation of alcoholic liver diseases or non-alcoholic steatohepatitis need to be expanded in subsequent studies. Third, we did not consider the relationship between genomics and radiomic features, where genomics could help to explain the significance of radiomics findings. Lastly, we only included peritumoral margins of 5 mm and 10 mm, considering that smaller margins may not contain sufficient information or lack of clinical application value. Such as 2mm or 3mm were discussed in other studies, too small distances are difficult to use in radiography and surgical procedures.

## 5. Conclusion

Radiomic features of a 5-mm peritumoral region may provide a potential non-invasive biomarker for the preoperative prediction of the risk of early tumor recurrence for patients with a solitary HCC ≤5 cm in diameter. A fusion model that combines the radiomic features of the peritumoral region and postoperative pathology could contribute to the development of a policy to guide individualized treatment of HCC.

## Data availability statement

The original contributions presented in the study are included in the article/supplementary material. Further inquiries can be directed to the corresponding author.

## Author contributions

FW and MC: research design and conception. BD: data collection. W-PH and L-ML: imaging data collection. J-BG: review of the manuscript and data analysis. All authors contributed to the article and approved the submitted version.

## Funding

This research was supported by a Medical Science and Technology of Henan Province, Joint construction project grant (LHGJ20210344).

## Conflict of interest

The authors declare that the research was conducted in the absence of any commercial or financial relationships that could be construed as a potential conflict of interest.

## Publisher’s note

All claims expressed in this article are solely those of the authors and do not necessarily represent those of their affiliated organizations, or those of the publisher, the editors and the reviewers. Any product that may be evaluated in this article, or claim that may be made by its manufacturer, is not guaranteed or endorsed by the publisher.

## Abbreviations

MVI, microvascular infiltration; CT, computed tomography; HCC, hepatocellular carcinoma; AP, arterial phase; PVP, portal venous phase; VOI, volume of interest; Vper, volume peritumoral; LASSO, Least absolute shrinkage and selection operator; ROC, receiver operating characteristic curve; AUC, Area Under Curve; DCA, decision curve analysis; RVI, radiogenomic venous invasion; TTPVI, two-trait predictor of venous invasion.
